# Real‐time dose reconstruction for wedged photon beams: a generalized procedure

**DOI:** 10.1120/jacmp.v12i4.3538

**Published:** 2011-11-15

**Authors:** A. Piermattei, F. Greco, A. Fidanzio, L. Azario, A. Porcelli, S. Cilla, D. Sabatino, A. Russo, G. D'Onofrio, M. Russo

**Affiliations:** ^1^ Istituto di Fisica Università Cattolica del S. Cuore Rome; ^2^ Unità Operativa di Fisica Sanitaria Università Cattolica del S. Cuore Rome; ^3^ Unità Operativa di Fisica Sanitaria, Centro di Ricerca e Formazione ad Alta Tecnologia nelle Scienze Biomediche Università Cattolica del S. Cuore Campobasso; ^4^ Unità Operativa di Radioterapia Marco Polo USI Rome; ^5^ Unità Operativa di Radioterapia Ospedale Spirito Santo Pescara; ^6^ Unità Operativa di Radioterapia Ospedale Belcolle Viterbo Italy

**Keywords:** *in vivo* dosimetry, real time, generalized procedure, quality assurance, wedged photon beams

## Abstract

A practical and accurate generalized procedure to reconstruct the isocenter dose $D_{\text{iso}}$ for 3D conformal radiotherapy (3DCRT) has been developed for X‐ray open beams supplied by linacs of different manufacturers and equipped with aSi electronic portal imaging devices (aSi EPIDs). This paper reports an extension of the method, to be applied at the wedged X‐ray beams characterized by the wedge attenuation factor $W_{\text{AF}}$.

Using water‐equivalent solid phantoms (SPs) of different thicknesses, w, and photon square fields of sizes, L, the generalized midplane doses $D^{0}(W_{\text{AF}},w/2,L)$ and generalized transit signals $s_{t}^{0}(W_{\text{AF}},w,L)$ by 38 beams of six different linacs were determined. The generalized data were fitted by surface equations and used together with the information of the ‘record & verify’ network of the centers. In this manner, for every beam, the $D_{\text{iso}}$ reconstruction was obtained in about 25 seconds after the treatment.

To test the *in vivo* dosimetric procedure, six pelvic treatments that used conformed wedged beams were carried out with three linacs of different manufacturers. For every beam, the comparison between the reconstructed $D_{\text{iso}}$ and the $D_{\text{iso},\text{TPS}}$ computed by the TPS, resulted in an acceptable tolerance level of ±5%, estimated for this kind of treatment.

Generally the *in vivo* dosimetry methods that use EPIDs require: (i) a special effort for the dosimetric commissioning with SPs of different thicknesses, and (ii) extra time for the analysis of the EPID signals. The proposed procedure simplifies the commissioning step and supplies for Varian, Elekta, and Siemens linacs equipped with the aSi EPIDs a quasi‐real time *in vivo* dosimetry for open and wedged 3DCRT fields.

PACS number: 87.53Xd

## I. INTRODUCTION

Recently, overdosages of patients due to the absence of adequate quality controls have been discovered in some radiotherapy centers of the United States of America and reported by the media.^(^ ^1^ ^)^ Professional training and dosimetric controls could have avoided these accidents. In particular, the *in vivo* dosimetry could discover the discrepancies between the reconstructed and the expected doses due to pretreatment and intreatment errors. The *in vivo* dose verification is actually one of the major concerns in radiotherapy, and we believe it will become mandatory in many countries in the future.^(^ ^2^ ^)^ Some researchers have demonstrated the advantages of reconstructing the delivered dose during the treatment using a 2D array as the amorphous silicon electronic portal imaging device (aSi EPID).^(^ ^3^ ^)^ The present authors have developed an *in vivo* dosimetry method for the 3D conformed radiotherapy technique (3DCRT) for open beams based on the ratios between the transit signals measured by aSi EPIDs, and the midplane doses of a water‐equivalent solid phantom (SP).^(^ ^4^ ^)^ The method has been applied to test head, thorax, pelvic, and breast tumors in radiotherapy treatments.^(^ ^4^ ^,^ ^5^ ^)^

However all the procedures based on the transit signals by the EPIDs^(^ ^6^ ^,^ ^7^ ^,^ ^8^ ^)^ require specific measurements with SPs for every beam. Recently, the authors have developed a generalized procedure for the *in vivo* dosimetric reconstruction at the isocenter point of the 3DCRT that used open photon beams of different linacs characterized by the ${\text{TPR}}_{20,10}$ quality index (herein named TPR). The method can be easily commissioned for linacs manufactured by Varian, Elekta, and Siemens and equipped with aSi EPIDs^(^ ^9^ ^)^ reducing the measurements with SPs.

The aim of this work was the implementation of the generalized procedure for the 3DCRT wedged beams characterized by the wedge attenuation factor, $W_{\text{AF}}$. A dedicated software that uses the ‘record and verify’ (R&V) network of the center supplied the *in vivo* dosimetry tests in quasi‐real time.

## II. MATERIALS AND METHODS

### A. Wedged beams

Table 1 reports same geometric and dosimetric characteristics of the 38 wedged X‐ray beams examined. The beams of 6, 10, and 15 MV were supplied by six linacs operating in five centers:

- two Clinac Varian linacs (Varian Medical System, CA), one supplying 6 MV and 10 MV and one supplying 6 MV and 15 MV
- two Elekta Precise linacs (Elekta, Stockholm, Sweden) supplying 6, 10 and 15 MV
- two Oncor Siemens (Siemens A.G. Erlangen, Germany) one supplying 6 MV, and 10 MV and one supplying 6MV and 15 MV

**Table 1:** Some characteristics such as the nominal megavoltage, wedge angle, and MU/min of the linac beams by Varian, Elekta, and Siemens. All the linacs used aSi EPIDs. The range MU/min used in clinical routine in the centers are underlined.

*Linacs*	*Clinac 2100 Varian*						*Elekta Precise*					*Siemens Oncor*			
Nominal Megavoltage (MV)		6	10		15		6		10		15	6	10		15
Range MU/min	100	200	300	400 ^a^	500	600	80	160	240	360	400	50	200	300	500
Wedge Angle (°)		15	30	45	60				60			15	30	45	60
Wedgep Position	external						internal					external			
aSi‐EPIDs Pixel Resolution Pitch (μm)	aS1000 1024×768 392			aS500 512×384 784			XRD 1640 AL5 1024×1024 400					OptiVue 1000ST 512×512 800			
Plate material Thickness Plate (mm)	Copper 1						Copper 1					Aluminum 1			
SED range (cm)	110–160						158.2–159.5					115–160			

a The range MU/min used in clinical routine in the centers are underlined.

The linacs were equipped with aSi EPIDs, based on panels of aSi sensors operating as a two‐dimensional photodiode array. A more detailed description of the functionality and basic properties of such devices is reported in the literature.^(^ ^10^ ^,^ ^11^ ^)^ The EPIDs included a metal plate that provides some buildup for the photons, and absorbs enough low energy scattered radiation that reduces image quality. A common source‐EPID metal plate distance (SED) equal to 159 cm was used in this work for every linac. The linacs were equipped with multileaf collimators and the X‐ray beams have been calibrated in water‐phantom following the IAEA protocol.^(^ ^12^ ^)^

**Figure 1:**
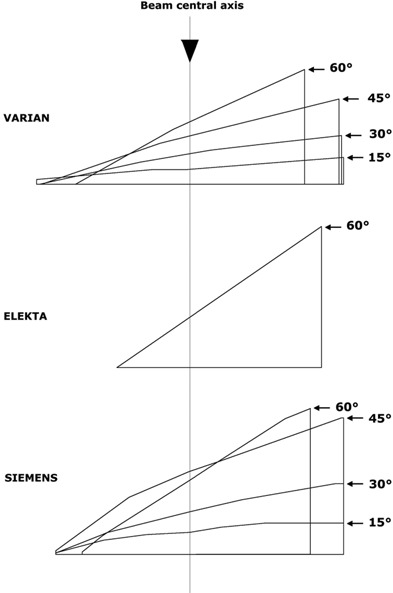
Central sections of the different wedges supplied by Varian for 15°, 30°, 45°, and 60°, by Siemens for 15°, 30°, 45°, and 60°, and by Elekta for 60°.

Figure 1 shows the central sections of the wedge filters supplied by the three manufactures for their linacs.

**Figure 2:**
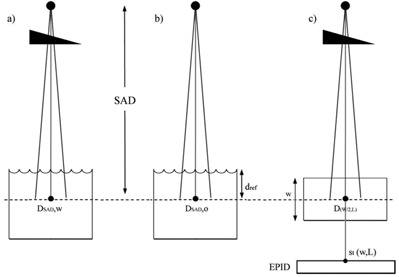
Dose at the SAD and $D_{\text{SAD},W}$ (a) determined for a wedged beam with the ion chamber at the reference depth $d_{\text{ref}}=10\;\text{cm}$ in water phantom and field size $10\times 10\;{\text{cm}}^{2}$; as in Fig. 2(a), the dose $D_{\text{SAD},0}$ was measured in water phantom for the open beam (b); measurements (c) of the dose D(w/2L) at the SP midplane and the transit signal $s_{t}(w,L)$ by the EPID using a square field size L.

The wedged beams were characterized by the wedge attenuation factor^(^ ^13^ ^)^ $W_{\text{AF}}$, defined for a reference beam $10\times 10\;{\text{cm}}^{2}$ at the SAD as the ratio between the doses obtained by an ion chamber at the reference depth $d_{\text{ref}}=10\;\text{cm}$ in water phantom, measured with the filter (Fig. 2(a)) $D_{\text{SAD},W}$ and without filter $D_{\text{SAD},0}$ (Fig. 2(b)).

(1) $$W_{\text{AF}}=\frac{D_{\text{SAD},w}}{D_{\text{SAD},0}}$$

Generally this factor is obtained during the commissioning measurements in water phantom.

However, in this work the index TPR^(^ ^12^ ^)^ for each wedge beam was also measured.

Table 2 reports some parameters that characterize the wedged beams supplied by (a) Varian, (b) Siemens, and (c) Elekta linacs. In particular, as a function of the nominal potential MV Table 2 reports, the wedge angles (defined as the angle between the beam central axis and the isodose direction at the $d_{\text{ref}}$, the thickness, t, of the filtered material on the beam central axis, $W_{\text{AF}}$, and the TPR indexes for open and wedged beams.

**Table 2:** Some characteristics of the wedged beams of 6, 10, and 15 MV supplied by linacs (a) Varian, (b) Siemens, and (c) Elekta. In particular, are reported the wedge angles, the wedge central thicknesses, t, the parameters $W_{\text{AF}}$, and the TPR index for open (wedge angle=0) and wedged beams.

a)															
*Nominal Megavoltage (MV)*			*6*					*10*					*15*		
Wedge Angle (°)	0	15	30	45	60	0	15	30	45	60	0	15	30	45	60
t (mm)	–	6	12	20	25	–	6	12	20	25	–	6	12	20	25
$W_{\text{AF}}$	–	0.775	0.620	0.492	0.403	–	0.804	0.668	0.521	0.428	–	0.819	0.684	0.523	0.429
TPR	0.668	0.677	0.682	0.689	0.695	0.738	0.740	0.744	0.745	0.739	0.762	0.767	0.770	0.765	0.767
b)															
*Nominal Megavoltage (MV)*			*6*					*10*					*15*		
Wedge Angle (°)	0	15	30	45	60	0	15	30	45	60	0	15	30	45	60
t (mm)	–	9	16	34	30	–	9	16	34	30	–	9	16	34	30
$W_{\text{AF}}$	–	0.692	0.530	0.323	0.357	–	0.735	0.588	0.386	0.418	–	0.752	0.607	0.404	0.438
TPR	0.675	0.685	0.691	0.700	0.695		0.747	0.755	0.763	0.760	0.768	0.773	0.773	0.774	0.770
c)															
*Nominal Megavoltage (MV)*			*6*					*10*					*15*		
Wedge Angle (°)		0		60			0		60			0		60	
t (mm)		–		24.6			–		24.6			–		24.6	
$W_{\text{AF}}$		–		0.276			–		0.287			–		0.286	
TPR		0.683		0.703			0.730		0.735			0.759		0.760	

In previous papers,^(^ ^14^ ^–^ ^16^ ^)^ the quality index TPR resulted a good parameter to select the generalized dose at midplane phantom and the transit signals. Indeed, these data showed for every couple of phantom thicknesses, w, and photon square field side, L, a proportional trend as a function of the TPR. The presence of the wedged filters for beams of the same MV reduces the soft radiation component and TPR values for open and wedged beams changed within 4%, 1.5%, and 0.7% for 6, 10, and 15 MV beams, respectively, confirming literature data.^(^ ^17^ ^)^ However, the TPR values for 15° and 60° filters change within 2.5%, 1.0%, and 0.5% for 6, 10, and 15 MV beams, respectively. It is important to underline that the TPR indexes are obtained as the ratios between the doses (at distance of 100 cm from the source) at depth 20 cm $\left(D_{20}\right)$ and 10 cm $\left(D_{10}\right)$ for open beams $10\times 10{\text{cm}}^{2}$ indicating the different beam hardness. For wedged beams, the filter shapes (Fig. 1) are projected to obtain modulated fluence with a minimum variation of the percentage depth dose along the beam central axis. In particular, the TPR index for wedged beams takes into account the hardening and the spatial photon fluence modulation, loosing the proportional trend with filter thickness, t. In this regard, TPRs for the wedged beams did not correlate with the dose at the mid plane phantom and the transit signals. The index $W_{\text{AF}}$ changes about 180% for the different wedge angles (Table 2), and it is well correlated with the doses and transit signals. For this reason the $W_{\text{AF}}$ was selected in this work to characterize the wedge beams.

### B. Isocenter dose reconstruction method

The method used for the dose reconstruction at the isocenter point $D_{\text{iso}}$ has been described in previous papers for open beams.^(^ ^14^ ^–^ ^16^ ^)^ Following that approach, correlation ratios between the transit signals, measured by an aSi EPID at the SED, positioned below an SP of thickness, w, and the dose values at the SP midplane along the beam central axis at the SAD (Fig. 2(c)) were determined for each beam of the same MV. In particular, for the wedged X‐ray beams of square field size L, specific correlation ratios $F(W_{\text{AF}},w,L)$ were determined by:

(2) $${F(W}_{\text{AF}},w,L)=\frac{s_{t}{(W}_{\text{AF}},w,L)}{{D(W}_{\text{AF}},w/2,L)}$$

where $s_{t}(W_{\text{AF},}w,L)$ are the transit signals and the $D(W_{\text{AF}},w/2,L)$ is the midplane dose in SP.

Moreover, a set of measurements were carried out positioning the phantom midplane (at distances d up to ±7 cm) below and above the SAD. This produced the different scattered photon contributions on the EPID due to the different distances between the portal detector and the bottom surface of the phantom that were taken into account by the ratios:

(3) $${f(W}_{\text{AF}},w,L,d)=\frac{s_{t}{(W}_{\text{AF}},w,L)}{s_{t}{(W}_{\text{AF}},w,L,d)}$$

These ratios also take into account the eventual changes of beam hardening due to the presence of the patient along the beam central axis that can be responsible for an aSi detector reading dependence.

#### B.1 Midplane doses by wedged fields

The SP, an RMI model 457 (Gammex, RMI Middelton, WI) with 30 cm square slabs of various thicknesses, presented a density of $1.045\pm 0.005\;g/{\text{cm}}^{3}$. A water equivalency correction factor, $k_{E}$ ^(^ ^18^ ^)^ for the SP resulted equal to $k_{E}=1.011$ ^(^ ^14^ ^)^ so the ion chamber reading in SP was multiplied by the $k_{E}$ before the midplane dose determination.

The midplanes of the SPs of different thicknesses, w, equal to 10, 22, 30 and 42 cm, were positioned at the SAD and irradiated with six square field sizes, L=4,8,10,12,16, and 20 cm (Fig. 2(c)). The midplane doses $D(W_{\text{AF},}w/2,L)$ measured in the five centers were carried out following the IAEA report^(^ ^12^ ^)^ and using the same cylindrical ion chamber, a PTW model 31010 type Farmer (PTW‐Freiburg, Germany), and a PTW Tandem (PTW‐Freiburg, Germany) as electrometer. One hundred MUs were delivered for each measurement.

However, MU calibrations were carried out in the centers using different source water‐phantom surface distances (SSD) or different dose values at the reference depth, $d_{\text{ref}}=10\;\text{cm}$ in water phantom. In particular, using an open $10\times 10\;{\text{cm}}^{2}$ field at the SSD, a reference output factor of 1 cGy/MU was assigned at the depth of the maximum dose, $d_{\max}$, for the Varian and Siemens linacs, while for the Elekta linacs the reference output factor was assigned at $d_{\text{ref}}$. For this reason, a factor $k_{0}$ has been defined as

(4) $$k_{0}=\frac{1\text{cGy}/\text{MU}}{D_{\text{SAD},w}}$$

In this way, the midplane doses $D(W_{\text{AF},}w/2,L)$ were normalized by the factor $k_{0}$

(5) $$D^{0}(W_{\text{AF}},w/2,L)=D(W_{\text{AF}},w/2,L)\cdot k_{0}$$

thus obtaining a set of generalized dose values $D^{0}(W_{\text{AF}},w/2,L)$ in terms of cGy/MU that were independent of the MU calibration adopted by the centers.

#### B.2 EPID calibration and transit signal measurements

The EPID images were exported as Digital Imaging Communication in Medicine (DICOM) files to be analyzed. Some characteristics of the aSi EPIDs and their running acquisition modes have been well reported in previous works for the open beams^(^ ^14^ ^–^ ^16^ ^)^ where the EPID signal, s, on the beam central axis in terms of arbitrary units (a.u.) was obtained by the average of the signals supplied by a number of central pixels around the beam's central axis for a $4\times 4\;{\text{mm}}^{2}$ area.

It is well known that aSi EPIDs present a nonlinearity response with the treatment exposure times due to the combination of the ‘image lag’ and the ‘gain ghosting’ effects. As reported by Mc Dermott et al.,^(^ ^10^ ^)^ to assess the linearity response of the different aSi EPIDs, the signals were measured delivering a number, N, of MUs equal to 5, 20, 50, 100, 200, 300, and 400 MU.

A correction factor for the linearity, $k_{\text{lin}}$, was determined for each clinical MU rate utilized (Table 1), as

(6) $$k_{\text{lin}}=\frac{s}{s_{N}}$$

where $s_{N}$ and $s_{N}$ were the signals obtained for 100 and N MU, respectively. Moreover, the signal dependence on the dose rate was analyzed by the $k_{\text{lin}}$ factors, obtained irradiating SPs with different thickness, w=10,22,30,42 cm (Fig. 2(c)).

The EPIDs were calibrated determining a reference transit signal, $s_{r,t}$, (in terms of arbitrary units a.u.) in the configuration reported in Fig. 2(c). In particular, the SP with a thickness w=22 cm was irradiated by a field $10\times 10\;{\text{cm}}^{2}$, with 100 MU and the $s_{r,t}$ value in a.u./MU was converted to one centi‐calibration unit per MU (1 cCU/MU). This way 38 sensitivity factors, $k_{s}$, in terms of cCU/a.u were determined for every EPID and wedged beam by:

(7) $$k_{s}=\frac{1\text{cCU}/\text{MU}}{s_{r,t}}$$

Thus, irradiating the SP of different thicknesses, w, with wedge beams of field sizes, L, the transit signals $s_{t}(W_{\text{AF},}w,L)$ in a.u./MU were multiplied by the $k_{s}$ factors, obtaining the generalized transit signals

(8) $$s_{t}^{0}(W_{\text{AF}},w,L)=s_{t}(W_{\text{AF}},w,L)\cdot k_{s}$$

The $s_{t}^{0}(W_{\text{AF}},w,L)$ values in terms of cCU/MU resulted independently from the MU calibration and the aSi EPID sensitivity. Of course, an integral transit signal, $s_{t}$(a.u.) (obtained by a number of MUs) multiplied by $k_{s}$ can be read in terms of cCU and resulted independent from the EPID sensitivity and the MU calibration.

The measurements of the $s_{t}^{0}(W_{\text{AF}},w,L)$ were also carried out, positioning the phantom midplane below and above the SAD (at distances, d, up to ±7 cm) as a function of w, and L. These last data were used to determine the generalized ratios, $f(W_{\text{AF},}w,L,d)$, defined by Eq. (3).

#### B.3 An interactive software for the generalized dose reconstruction

Using a commercial software package, TableCurve 3D, SPSS‐Science2000 (Systat Software, Inc., San Jose, CA),^(^ ^19^ ^)^ the data reported by Eqs. (5) and (8) were analyzed and fitted by surface equations. The software package automates the surface‐fitting process minimizing the sum of squares of the residuals (where a residual is simply the difference between the experimental value and the one computed from the surface‐fit equation).^(^ ^20^ ^)^ This way, the surface equations for the $s_{t}^{0}(W_{\text{AF}},w,L)$ and the $D^{0}(W_{\text{AF}},w/2,L)$ experimental data can be used to obtain generalized correlation ratios (in terms of CU/Gy, Eq. (2)).

In this work, a dedicated software DISO that uses all the information stored in the R&V system of the center has been used to supply the dose reconstruction in quasi‐real time. DISO is configured by the user introducing the dosimetric parameters of the wedged beams $\left(W_{\text{AF}},k_{0}\right)$ and of the EPIDs $\left(k_{s},k_{\text{lin}}\right)$. DISO has been developed in two modules: the first one for the pretreatment step where the DICOM files supplied by the CT scanner and the TPS can be used to obtain the equivalent square field, the water‐equivalent thicknesses, w and the depth $w_{\text{iso}}$, independently from the TPS.

The second module of the DISO is for the ‘posttreatment step’. The DICOM files from the EPID after the patient's daily treatment have been analyzed to determine, for every beam, the $D_{\text{iso}}$ value, as well as the ratio ${R=D}_{\text{iso}}/D_{\text{iso},\text{TPS}}$. In particular, for every beam, the images of the isocenter CT scan with the beam geometrical edges and the central axis direction have been reported.

A preliminary test to verify the accuracy of the generalized procedure was obtained following 12 radiotherapy treatments of prostatic tumors, four cases for each of the three linacs (Varian, Elekta, and Siemens) (not included in Table 1). This meant that the factors $k_{0}$, $k_{s}$, $k_{\text{lin}}$ and $W_{\text{AF}}$ of the new linacs were determined. The selection of these pelvic treatments assured a good accuracy in terms of: (i) the $D_{\text{iso},\text{TPS}}$ computation in quasi‐homogeneous tissues, and (ii) the patient setup. For each patient, six tests were carried out for a total of 144 tests for wedge beams of 10 and 15 MV used for the later‐lateral irradiations. In particular, the 15 MV Siemens beams used 30° and 45° wedges, while the 10 MV Varian beams used a 30° wedge, and the 10 MV Elekta beams used the 60° wedge.

The TPSs used for the MU computations to deliver the $D_{\text{iso},\text{TPS}}$ were two 3D Eclipse (Eclipse 7.3.10 Varian, Palo Alto CA, USA) and one 3.0 Oncentra Masterplan (Nucletron BV, Venendal, The Netherlands) in the center that used the Elekta linac.

The aim of these 144 tests was to verify the ratios, R, obtained by the generalized procedure and that were within the tolerance level 5%, estimated for this treatment.

## III. RESULTS

### A. Midplane doses and transit signals

Table 3 reports the factors $k_{0}$ and $k_{s}$ determined for 15 of the 38 wedged beams. The $W_{\text{AF}}$ long‐term stability was estimated equal to ±0.3% — that means within the experimental uncertainty. A long‐term stability for the $s_{r,t}$ was equal to 2% (2 SD). This means that when the $s_{r,t}$ changes in time over this tolerance level, a new $k_{s}$ factor should be adopted to take into account the change of the EPID sensitivity.

**Table 3:** $k_{0}$ and $k_{s}$ factors determined for 15 beams (supplied by three linacs) of the 38 wedged beams examined in this work.

	*Clinac 2100 Varian*					*Elekta Precise*			*Siemens Oncor*					
Nominal Mega Voltage (MV)	6		10		15		6	10	15	6		10		15	
Wedge Angle (°)	15	60	15	60	15	60	60	60	60	15	60	15	60	15	60
$k_{0}$	1.63	3.13	1.42	2.67	1.33	2.53	3.01	2.89	2.90	1.77	3.44	1.85	3.25	1.43	2.45
$k_{s}\cdot 10^{-6}(\text{cCU}/a.u)$	7.79	14.72	6.36	12.22	5.58	10.63	8.75	6.36	6.12	3.05	6.68	2.74	4.21	2.36	4.05

The $k_{\text{lin}}$ factors for the same EPID model resulted within ±0.5% independent of the nominal MV and the dose rate, in agreement with the data reported by McDermott et al.^(^ ^10^ ^)^ Table 4 reports the average values of the $k_{\text{lin}}$ factors determined for the aSi EPID models here used (Table 1).

**Table 4:** $k_{\text{lin}}$ factors determined by Eq. (6) for Varian, Elekta, and Siemens linacs. The data have been obtained by averaging the $k_{\text{lin}}$ factors determined by different quality beams and different dose rates.

	*5 MU*	*20 MU*	*50 MU*	*100 MU*	*200 MU*	*300 MU*	*400 MU*
Varian (UCSC)	1.020	1.010	1.005	1.000	0.997	0.995	0.994
Elekta (UCSC)	1.020	1.011	1.008	1.000	0.997	0.995	0.994
Siemens (VT)	1.029	1.016	1.007	1.000	0.993	0.990	0.989

**Figure 3:**
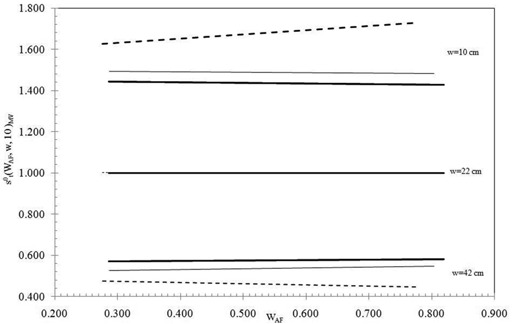
Linear fits by the experimental $s_{t}^{0}{\left(W_{\text{AF},}w,10\right)}_{\text{MV}}$ values (obtained with a field $L=10\times 10{\text{cm}}^{2}$) as function of $W_{\text{AF}}$, for the 6 MV (dotted line), 10 MV (thin line), and 15 MV (heavy line) beams. The fits reported here were obtained by SP thicknesses w=10,22, and 42 cm and using $10\times 10\;{\text{cm}}^{2}$ fields.

Figure 3 reports the linear fits of the generalized transit signals $s_{t}^{0}{\left(W_{\text{AF},}w,10\right)}_{\text{MV}}$ for $10\times 10\;{\text{cm}}^{2}$ fields of the same MV as a function of the $W_{\text{AF}}$ and for phantom thicknesses w=10,22, and 42 cm. The fits reproduce the measured data, well within the experimental uncertainties ±2.5% (2SD) estimated for these data, and the same accuracy was obtained for the other field sizes. Figure 3 shows that the generalized signals for w=22 cm are equal to the unity for all the MV beams (Eq. (8)). Of course, increasing or decreasing the phantom thickness w, data result, respectively, less or greater than the unity and, in particular, the changes from the unity increase for beams of minor MV values. As reported in Table 2, the poor variations of the TPRs for wedged beams of the same MV, (in particular, for 10 MV and 15 MV) is coherent with the small variations of the relative photon transmissions given by Eq. (8). For example, the heavy line for 15 MVs reported in Fig. 3 for the w=42 cm is obtained by the ratios

(9) $$s_{t}^{0}{\left(W_{\text{AF}},42,10\right)}_{15}=\frac{s_{t}(W_{\text{AF}},42,10)}{s_{t}(W_{\text{AF}},22,10)}$$

and for different $W_{\text{AF}}$ values, these ratios are constant enough, while for the beams of 6 MV, the ratios change for the different wedges.

The generalized experimental transit signals $S_{t}^{0}{\left(W_{\text{AF}},w,L\right)}_{\text{MV}}$ (Eq. (8)) were fitted by surface equations, once for every square field, L, by:

(10) $$s_{t,\text{MV}}^{0}=b_{1}+b_{2}W_{\text{AF}}+b_{3}w+b_{4}W_{\text{AF}}^{2}+b_{5}w^{2}+b_{6}W_{\text{AF}}w+b_{7}W_{\text{AF}}^{3}+b_{8}w^{3}+b_{9}W_{\text{AF}}w^{2}+b_{10}W_{\text{AF}}^{2}w$$

where the ten adjustable coefficients, $b_{i}(i=1,\ldots ,10)$ are real numbers obtained through the fitting procedure.

Similar trends as from Fig. 3 were obtained for the midplane dose $D^{0}{\left(W_{\text{AF}},w/2,L\right)}_{\text{MV}}$ and the linear fits reproduced the experimental data well within the experimental uncertainty ±2% (2SD) estimated for the generalized doses. In conclusion, for every MV, the experimental doses $D^{0}{\left(W_{\text{AF},}w/2,L\right)}_{\text{MV}}$ (Eq. (5)) were fitted by surface equations once for every square field L by:

(11) $$D_{\text{MV}}^{0}=a_{1}+a_{2}W_{\text{AF}}+a_{3}w+a_{4}W_{\text{AF}}^{2}+a_{5}w^{2}+a_{6}W_{\text{AF}}w$$

where the six adjustable coefficients, $a_{i}(i=1,\ldots ,6)$, are real numbers obtained through the fitting procedure. The number of adjustable parameters (10 and 6 in Eqs. (10) and (11)) were chosen to obtain the computed data within the uncertainties of the experimental data estimated for the midplane doses and the transit signals.

**Figure 4:**
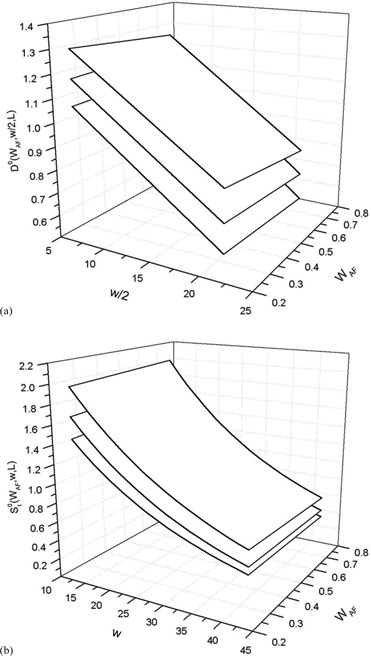
Three surfaces (a) that fitted the $s_{t}^{0}{\left(W_{\text{AF},}w,10\right)}_{\text{MV}}$ data for the square field width L=4,10, and 20 cm from the bottom to the top; three surfaces (b) that fitted the $s_{t}^{0}(W_{\text{AF},}w,L)$ data for square field sides 4, 10, and 20 cm from the bottom to the top.

Figure 4 reports, for the 6 MV X‐ray beams with L=4,10, and 20 cm, the surfaces (Eq. (11)) that fitted the experimental data $D^{0}{\left(W_{\text{AF}},w/2,L\right)}_{\text{MV}}$ (Fig. 4(a)) and the fitting surfaces (Eq. (10)) for the experimental data $s_{t}^{0}{\left(W_{\text{AF}},w,L\right)}_{\text{MV}}$ (Fig. 4(b)). The differences between the generalized experimental data and the computed data given by Eqs. (10) and (11) were, respectively, within 1.3% (2SD) with maximum residuals up to 1.8% and 2.0% (2SD) with maximum residuals up to 2.5%. This means that the generalized ratios

(12) $$F^{0}(W_{\text{AF}},w,L)=\frac{s_{t,\text{MV}}^{0}}{D_{\text{MV}}^{0}}$$

were within 3% equal to the ratios obtained by the experimental data.

**Figure 5:**
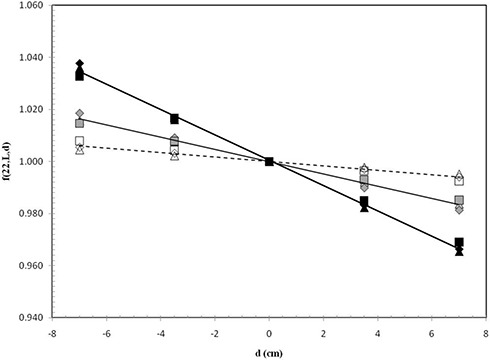
Averaged factors $f{\left(22,L,d\right)}_{6\text{MV}}$ for different $W_{\text{AF}}$ indexes obtained by the SP thickness of w=22 cm and using beams of 6 MV supplied by Varian (Δ), Elekta (□), and Siemens (⋄) linacs. The data are reported for L=4 cm (open symbol), L=10 cm (green symbol), and L=20 cm (black symbol).

The $f(W_{\text{AF},}w,L,d)$ resulted independently from the $W_{\text{AF}}$ (within 0.3%). Figure 5 shows for the w=22 cm the $f{\left(22,L,d\right)}_{6\text{MV}}$ factors (Eq. (3)) for the 6 MV wedged beams (supplied by the three linacs: Elekta, Varian, and Siemens) with the field sizes $4\times 4\;{\text{cm}}^{2},10\times 10\;{\text{cm}}^{2}$, and $20\times 20\;{\text{cm}}^{2}$. The $f{\left(22,\;L,\;d\right)}_{6\text{MV}}$ values were obtained averaging the ratios in Eq. (3) for different $W_{\text{AF}}$ indexes. This way, the $f{\left(w,L,d\right)}_{6\text{MV}}$ data were tabulated for each nominal MV parameter in terms of (w, L, d).

### B. Isocenter dose determination

Following in part the approach used for open beams,^(^ ^9^ ^)^ the dose $D_{\text{iso}}$ for a generic wedged beam has been obtained by:

(13) $$D_{\text{iso}}=s_{t}(W_{\text{AF}},w,L,d)\cdot \frac{k_{s}\cdot k_{\text{lin}}}{k_{0}}\cdot {\left[\frac{f(w,L,d)}{F^{0}(W_{\text{AF}},w,L)}\cdot {\text{TMR}}_{w/2}^{w_{\text{iso}}}\right]}_{\text{MV}}$$

where $s_{t}(W_{\text{AF},}w,L,d)$ in terms of a.u. is the EPID integral signal that is corrected by: (i) the sensitivity factor $k_{s}$ to take into account for the EPID sensitivity, (ii) by the $k_{0}$ factor to take into account for the MU calibration, and (iii) by the factor $k_{\text{lin}}$, to take into account for the nonlinearity of the EPID signal with the MUs. In square brackets, Eq. (13) reports, for every MV, the $F^{0}(W_{\text{AF},}w,L)$ correlation ratio (Eq. (12)), the $f{\left(w,L,d\right)}_{\text{MV}}$ factors and the tissue maximum ratios ${\text{TMR}}_{w/2}^{w_{\text{iso}}}$.^(^ ^21^ ^)^ In particular, by the $W_{\text{AF}}$ beam index and the patient's radiological thickness w (Eqs. (10) and (11)), supplied the data for the ratio $F^{0}(W_{\text{AF}},w,L)$, while the $f{\left(w,L,d\right)}_{\text{MV}}$ and the ${\text{TMR}}_{w/2}^{w_{\text{iso}}}$ ratios were selected by interpolations of tabulated data for each MV parameter.

The dosimetric accuracy of the method was analyzed in a previous paper^(^ ^4^ ^)^ and, in particular, for the ratio, R, between the *in vivo* reconstructed dose $D_{\text{iso}}$ and the predicted dose, $D_{\text{iso},\text{TPS}}$, a tolerance level of 5% was assumed.^(^ ^5^ ^)^

**Figure 6:**
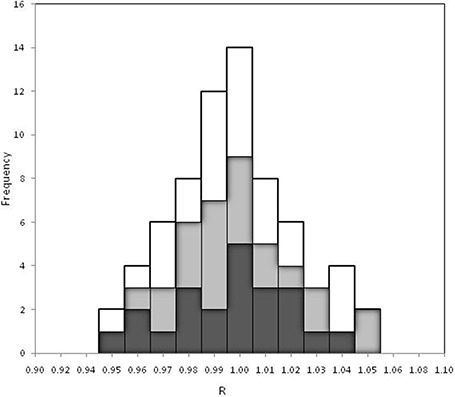
Histogram of 131 tests carried out for 12 pelvic treatments using wedged beams of 6 MV (black), 10 MV (grey), and 15 MV (white) supplied by three linacs (Varian, Elekta and Siemens).

Figure 6 reports the histogram of 131 tests over the 144 carried out for six pelvic treatments. In fact, 13 tests on three patients showed dose overestimations between 8% and 12% due to the presence of air pockets along the beam central axis, and these results were not reported in Fig. 6. In these last tests, the transit signal profiles that crossed the beam central axis showed small localized increases if compared with other previous controls. The visual portal imaging (VPIs) obtained before the treatment confirmed the presence of gas pockets. However, for every patient, the sum of doses (obtained by the beams reported in Fig. 6) were well within 4% of the stated doses, $D_{\text{iso},\text{TPS}}$. Figure 6 confirmed the ratios, R, well within the tolerance level of 5% estimated for each test of the 3DCRT pelvic treatment. Moreover, the generalized procedure that adopts a new software for the DISO, supplied the $D_{\text{iso}}$ reconstructions after 1.5 minutes at the end of a treatment with four beams.

## IV. DISCUSSION

The dosimetric procedure for the *in vivo* dosimetry here reported is based on the use of generalized functions $D_{\text{MV}}^{0}$ and $s_{t,\text{MV}}^{0}$ obtained fitting the experimental data of 38 wedged beams of linacs of different manufacturers. Original calibration procedures were adopted for the $D^{0}(W_{\text{AF},}w/2,L)$ (Eq. (5)) that resulted independently of the MU calibration adopted by the centers and the $s_{t}^{0}(\text{TPR},w,L)$ (Eq. (8)) that resulted independent of both the EPID sensitivity and the MU calibration of the beams.

The linacs, as the EPIDs manufactured by Varian, Elekta, and Siemens, present a different collimator and wedge filter assemblage^(^ ^22^ ^,^ ^23^ ^)^ (different distances from the source), as well as different EPID‐building.^(^ ^8^ ^,^ ^24^ ^,^ ^11^ ^)^ This can be a cause of different scatter photon contributions by collimators, wedges, and by the EPID itself. However, the effect of the different photon scattered contributions from the collimators and the wedges as a function of the beam size seems to be negligible, and is based on the low residuals for the fits of Eq. (11). In other words, the generalized $D^{0}{\left(W_{\text{AF},}w/2,L\right)}_{\text{MV}}$ seems to be independent of linac type. Moreover, the low residuals by Eq. (10) suggested that the effects of different backscatter contributions from the EPIDs of different manufacturers were negligible for the SP thicknesses and field sizes here used. In other words, the generalized $s_{t}^{0}{\left(W_{\text{AF}},w,L\right)}_{\text{MV}}$ values seemed to be independent of linac type.

The time required for the measurements with the SPs for the correlation ratios (Eq. (2)) and the ratios of Eq. (3) were estimated to be six hours per open and wedged beams of the same MV. Using the generalized functions, the measurements to be carried out in the centers were reduced to those for the determination of the $W_{\text{AF}}$, $k_{0}$, $k_{s}$, and $k_{\text{lin}}$ parameters. We have verified that in 2 hours it is possible to determine the four parameters for all the linac beams. In particular, Eq. (7) supplies the $k_{s}$ factors obtained with w=22 cm of SP. At the moment, we have determined for each beam of the different linacs, the ratios between $k_{s}$ and $k_{s,0}$. The latter was obtained in the same manner but in the absence of the SP. In this way, the $k_{s}$ stability could be tested every month, determining the $k_{s,0}$.

The extra time needed for the pretreatment step resulted in about 40 sec per beam, while for the post‐treatment step, it resulted about 25 sec per beam.

## V. CONLUSIONS

*In vivo* dosimetry is today considered a special tool to avoid accidents,^(^ ^2^ ^)^ and many researchers are studying new methods based on the use of EPIDs that are easy to implement, simple, efficient in their daily use, and sufficiently accurate for the purpose they are serving.^(^ ^25^ ^)^ Recent methods based on transit dosimetry require specific measurements in phantom^(^ ^10^ ^,^ ^7^ ^,^ ^4^ ^)^ and data analysis after the treatment. This paper extends a generalized procedure developed for open beams at the 3DCRT wedged beams supplied by linacs manufactured by Varian, Elekta, and Siemens. This way, the implementation measurements in solid water phantoms can be strongly reduced and the method can be easily included in the QA program^(^ ^26^ ^)^ of the centers. Moreover, the dedicated software DISO supplies an accurate $D_{\text{iso}}$ reconstruction at the end of the fractioned therapy in quasi‐real time.

The method based on the correction functions can be applied for breast treatment^(^ ^27^ ^)^ using measurements in cylindrical water phantoms. This means that new generalized functions could be determined for this technique.

At the moment, the authors are studying the possibility of extending the generalized procedure for the $D_{\text{iso}}$ reconstruction for intensity‐modulated radiotherapy beams, in particular for a 2D *in vivo* dose investigation.

## ACKNOWLEDGEMENTS

This work was supported by the “Istituto Nazionale di Fisica Nucleare applicata alla Medicina (INFN‐MED)” project DISO (2011).
